# Hypoxia Potentiates Glioma-Mediated Immunosuppression

**DOI:** 10.1371/journal.pone.0016195

**Published:** 2011-01-20

**Authors:** Jun Wei, Adam Wu, Ling-Yuan Kong, Yongtao Wang, Gregory Fuller, Isabella Fokt, Giovanni Melillo, Waldemar Priebe, Amy B. Heimberger

**Affiliations:** 1 Department of Neurosurgery, The University of Texas M. D. Anderson Cancer Center, Houston, Texas, United States of America; 2 Department of Pathology, The University of Texas M. D. Anderson Cancer Center, Houston, Texas, United States of America; 3 Department of Experimental Therapeutics, The University of Texas M. D. Anderson Cancer Center, Houston, Texas, United States of America; 4 National Cancer Institute, Frederick, Maryland, United States of America; University of Colorado Denver, United States of America

## Abstract

Glioblastoma multiforme (GBM) is a lethal cancer that exerts potent immune suppression. Hypoxia is a predominant feature of GBM, but it is unclear to the degree in which tumor hypoxia contributes to this tumor-mediated immunosuppression. Utilizing GBM associated cancer stem cells (gCSCs) as a treatment resistant population that has been shown to inhibit both innate and adaptive immune responses, we compared immunosuppressive properties under both normoxic and hypoxic conditions. Functional immunosuppression was characterized based on production of immunosuppressive cytokines and chemokines, the inhibition of T cell proliferation and effector responses, induction of FoxP3+ regulatory T cells, effect on macrophage phagocytosis, and skewing to the immunosuppressive M2 phenotype. We found that hypoxia potentiated the gCSC-mediated inhibition of T cell proliferation and activation and especially the induction of FoxP3+T cells, and further inhibited macrophage phagocytosis compared to normoxia condition. These immunosuppressive hypoxic effects were mediated by signal transducer and activator of transcription 3 (STAT3) and its transcriptionally regulated products such as hypoxia inducible factor (HIF)-1α and vascular endothelial growth factor (VEGF). Inhibitors of STAT3 and HIF-1α down modulated the gCSCs' hypoxia-induced immunosuppressive effects. Thus, hypoxia further enhances GBM-mediated immunosuppression, which can be reversed with therapeutic inhibition of STAT3 and HIF-1α and also helps to reconcile the disparate findings that immune therapeutic approaches can be used successfully in model systems but have yet to achieve generalized successful responses in the vast majority of GBM patients by demonstrating the importance of the tumor hypoxic environment.

## Introduction

We and other investigators have recently demonstrated that the GBM-associated cancer stem cells (gCSCs) participate in tumor-mediated immunosuppression by both secreted and membrane-associated mechanisms and inhibit both innate and adaptive immunity [1], [2], [3], [4]. Specifically, gCSCs markedly inhibited adaptive T cell proliferation and activation and induced regulatory T cells by both direct cell-to-cell contact and secreted soluble factors in both donor and autologous T cell assays with similar results [2], [3]. Furthermore, the gCSCs also induce macrophage/microglia to assume an immunosuppressive M2 phenotype mediated by the phosphorylated signal transducer and activator of transcription (pSTAT3) pathway with impaired innate functions that could be blocked by WP1066 [4]. These findings conflict with those that suggest that the gCSC may be targeted using a variety of immunotherapeutic approaches [5], [6], [7]. The reconciliation of this disparity may reside in environmental factors that are minimized or negated in cell lines that are perpetuated *in vitro*. Because gCSCs are a heterogeneous cell population, and immunosuppressive properties provide no survival benefit in a cell-culture environment, selective pressures may favor the loss of immunosuppressive phenotypes over time. This important consideration may falsely lead an investigator to implement an immune therapeutic strategy based on preclinical data that has artificially minimized the influence of tumor mediated immunosuppression.

Hypoxic microenvironments are a frequent characteristic of GBM that are the consequences of morphologically and functionally inappropriate neovascularization, irregular blood flow, anemia, and the high oxygen consumption of rapidly proliferating malignant cells [8]. These hypoxic microenvironments are a powerful stimulus for the expression of genes involved in tumor cell proliferation and angiogenesis [9]. Tumor hypoxia can activate the pSTAT3 immunosuppressive pathway thereby triggering the downstream synthesis of hypoxia inducible factor (HIF)-1α that can then induce regulatory T cells [10] and vascular endothelial growth factor (VEGF)[11], [12], which in addition to promoting angiogenesis is also immunosuppressive [13], [14]. In addition, other glioma-elaborated immune suppressive cytokines such as transforming growth factor (TGF)-β [15], [16], [17], soluble colony stimulating factor (sCSF)-1 [18], [19], C-C chemokine ligand (CCL)-2 [20], [21] and galectin-3 [22], [23] have also been shown to be associated with or induced by HIF-1α and hypoxia [24], [25], [26], [27], [28].

The STAT3 pathway, which is induced in the various immune populations, has been shown to be a potent regulator of anti-inflammatory responses in both innate [29] and adaptive immunity [30], including immunosuppression potentiated by the gCSC [2], [4]. CNS macrophages (microglia), which are the dominant infiltrating immune cells in GBM and the principle mediators of innate immunity within the tumor [31], [32], become tumor-associated macrophages (TAMs) when exposed to the hypoxic tumor microenvironment (TAMs) [33], [34]. TAMs become polarized toward immunosuppressive and tumor supportive phenotypes (M2) via the STAT3 pathway and then contribute to angiogenesis and tumor invasion [26]. We have demonstrated that these TAMs, which arise from circulating monocytes that are recruited into the tumor microenvironment [35], can be modulated by the gCSCs by inducing an immunosuppressive phenotype via the STAT3 pathway[4]. Since HIF-1α can promote the expansion of CD133-positive gCSCs [36], [37], it is possible that hypoxia induces a feed-forward mechanism of tumor-mediated immunosuppression.

We suspected that hypoxia potentiates the ability of the gCSC to evade immune surveillance that could account for, in part, the discrepancy between investigators that demonstrate immunological clearance of this population [5], [6], [7] and the grim reality of recurrence and failure of gCSC eradication even in the setting immune therapeutic approaches. *We therefore hypothesized that hypoxia would further potentiate the innate and adaptive immunosuppressive properties of gliomas which could be reversed by inhibiting hypoxia induced pathways such as pSTAT3*. *T*o determine whether we could reverse the gCSC-mediated immunosuppressive effects of hypoxia, we tested the pSTAT3 and HIF-1α inhibitor WP1066 [38] and WP744, an anthracycline compound [39] with suspected HIF-1α inhibitory effects [40].

## Materials and Methods

### Ethical Treatment of Research Subjects and Patient Consent

Each patient provided written informed consent for tumor tissues, and this study was conducted under protocol #LAB03-0687, which was approved by the institutional review board of The University of Texas M. D. Anderson Cancer Center.

### Derivation of human gCSCs

GBM specimens were processed within 4 hours after resection. The four separate gCSCs used in this study were isolated from four different individual GBM patients. They were washed with DMEM-F-12 medium, disassociated as previously described [41], and have been previously characterized [2], [3], [4]. The gCSCs were grown in neurosphere medium (DMEM-F-12 medium containing 20 ng/ml of epidermal growth factor, basic fibroblast growth factor (Sigma, St. Louis, MO), and B27 (1∶50; Invitrogen, Carlsbad, CA). To minimize possible phenotypic changes in gCSCs due to prolonged maintenance in vitro and multiple passages, early passage gCSCs were frozen in neurosphere medium containing 5% DMSO and stored at −70°C. After thawing, gCSCs were used in experiments after 1 or 2 passages.

### Hypoxia induction

Dissociated gCSCs (1.5×10^6^ cells) were plated in T-25 flasks in 4 ml of neurosphere medium and cultured under normoxic conditions for 48 h to allow the cells to re-enter log-growth phase. The cells were then moved to a hypoxia chamber (Sanyo Manufacturing) at 1% O_2_ for another 24 h. Alternatively, cells were treated with 100 µM of hypoxia-mimic chemical desferrioxamine mesylate (DFX; Sigma).

### Antibodies and reagents

Tissue-culture grade monoclonal antibodies to CD3 (OKT3) and CD28 (28.6) were obtained from eBioscience. The cell surface was stained with phycoerythrin (PE), fluorescein isothiocyanate (FITC), or allo-phycocyanin-conjugated (APC) antibodies against the following proteins: CD3, CD4, CD8, major histocompatibility complex (MHC) I, MHC II, CD40, CD80, CD86, and B7-H1 (BD Pharmingen, San Diego, CA) and CD133 (Miltenyi Biotech, Auburn, CA). To detect intracellular cytokines, PE-conjugated antibodies against interleukin (IL)-2 and interferon (IFN)-γ (R&D Systems) were used. To detect pSTAT3, PE-conjugated antibodies against pSTAT3 (BD Pharmingen, San Diego, CA) were used. Appropriate isotype controls were used for each antibody.

The inhibitor agents WP1066 and WP744 were synthesized and supplied by Waldemar Priebe. WP1066 was stored as a 10 mM stock solutions in dimethyl sulfoxide and diluted with PBS and WP744 was stored as a 10 mM stock solutions in N,N-dimethylacetamide. WP1066 is a specific and potent inhibitor of STAT3 [38] and of down-stream STAT3-regulated products (such as HIF-1α) that achieves *in vivo* physiological doses of 2 µM, including within the central nervous system [42]. WP744 is an anthracycline compound [39] that is suspected to inhibit HIF-1α [40] and achieves *in vivo* physiological doses of 0.1 µM (unpublished data). Only WP1066 possesses significant pSTAT3 inhibitory activity. The cell viability of the gCSCs based on MTT (dimethyl thiazolyl diphenyl tetrazolium) assays is not affected by doses of 2 and 0.1 µM of WP1066 or WP744, respectively.

### ELISA

Supernatant medium from the gCSC cell cultures were assayed for cytokine concentrations using ELISA kits as described by the manufacturer (R&D Systems). These supernatants were collected from 3×10^6^ cells after 5 d in culture and stored at −20°C. The supernatants were added in duplicate to appropriate pre-coated plates. After the plates were washed, horseradish peroxidase-conjugated detection antibody was added. The substrate used for color development was tetramethylbenzidine. The optical density was measured at 450 nm with a microplate reader (Spectra Max 190; Molecular Devices, Sunnyvale, CA), and chemokine concentrations were quantified using SoftMax Pro software (Molecular Devices). The detection limits for CC chemokine ligand 2 (CCL-2) were 5 pg/ml; TGF-β1, 16 pg/ml; IL-10, 5 pg/ml; soluble colony-stimulating factor (sCSF)-1, 8 pg/mL; VEGF, 5 pg/ml; Galectin-3, 10 pg/ml; and macrophage inhibitory cytokine (MIC)-1, 8 pg/ml.

### Human peripheral blood mononuclear cells (PBMCs)

PBMCs were prepared from healthy donor blood (Gulf Coast Blood Center, Houston, TX). CD14+ monocytes were purified from PBMCs by positive selection using CD14 microbeads (Miltenyi Biotech, Auburn, CA), and CD3+ T cells were purified from PBMCs by negative selection using a Pan T Cell Isolation Kit II (Miltenyi Biotech, Auburn, CA), according to the manufacturer's instructions.

### Human monocyte assays

5×10^5^ CD14+ monocytes were incubated with 500 µL (1×10^6^ cells/mL) of supernatant medium from gCSCs cultured in both normoxic and hypoxic conditions, as well as in neurosphere medium as controls. Induction of pSTAT3 was determined by flow cytometry after overnight incubation. Expression of the cell surface markers MHC I, MHC II, CD80, CD86, CD163, and CD204 was determined by flow cytometry after 48 h of incubation. After 72 h of incubation, monocytes were harvested for phagocytosis assays, and supernatants were collected for cytokine ELISA.

### Flow cytometry

FITC-conjugated anti-CD4 (RPA-T4) and APC-conjugated anti-CD8 (RPA-T8) antibodies were used for cell surface staining. Sub-analysis of the T cell populations was based on the gated surface expression of CD4 and CD8. To detect FoxP3 protein expression, the surface stained cells were further subjected to intracellular staining with PE-conjugated monoclonal antibodies to human FoxP3 (clone PCH101, eBiosciences) using staining buffers and conditions specified by the manufacturer. For intracellular cytokine staining, cells were stimulated for 6 h in the presence of 50 ng/ml phorbol myristate acetate (PMA), 500 ng/ml ionomycin (Sigma-Aldrich), and 2 µM monensin (GolgiStop, BD Sciences). Then the cells were incubated with FITC-conjugated anti-CD4 and APC-conjugated anti-CD8 (RPA-T8) antibodies for surface staining followed by intracellular staining using PE-conjugated anti-mouse IFN-γ (4S.B3) antibody and FIX/PERM buffers (BD Pharmingen) according to the manufacturer's instructions. For HIF1α intracellular staining, gCSCs were first fixed with cold 4% paraformaldehyde at room temperature for 10 minutes; following fixation, the cells were washed and permeabilized by 1% saponin on ice for 30 minutes, and then stained by APC-conjugated anti-human HIF-1α antibody (R&D Systems) for 45 minutes at room temperature in the dark. Intracellular p-STAT3 intracellular staining was performed as previously described [43]. Flow cytometry acquisition was done with a FACSCaliber (Becton Dickinson, San Diego, CA), and data analysis was with FlowJo software (TreeStar, Ashland, OR).

### Cell proliferation assay and regulatory T cell induction assay

The conditioned medium from the gCSCs was added to 2 µM CFSE-labeled 3×10^5^ PBMCs/ml in the presence of 1 µg/ml pre-bound anti-CD3/anti-CD28 antibodies. After 72 h, T cell proliferation was measured by division of CFSE-labeled cells as recorded with flow cytometry. The inhibition of T cell proliferation was represented by the percentage decrease of CSFE+ divided T cells compared to neurosphere medium control. To detect FoxP3+ regulatory T cells, CD4 surface staining and then FoxP3 intracellular staining were performed on immune cells cultured for 96 h.

### Phagocytosis assay

CD14+ monocytes were incubated with either normoxic or hypoxic gCSC-conditioned medium for 72 h. Positive control monocytes were incubated with neurosphere medium for 72 h. Cell viability was assessed by Trypan Blue exclusion, and live monocytes were transferred to a 96-well plate at a concentration of 1.0×10^5^ cells/well and incubated at 37°C in a humidified atmosphere containing 95% air/5% CO_2_ for 1 h to allow the cells to adhere. The cells were then incubated with pHrodo *E. coli* BioParticles (Invitrogen) suspended in PBS for 2 h at 37°C at atmospheric CO_2_ levels. Cells were examined using fluorescence microscopy, and phagocytosis was quantified by measuring the mean fluorescence intensity per cell and corrected for background using Adobe Photoshop CS software. Percent phagocytosis levels relative to the control were calculated for each experimental condition.

### Western Blot

After exposure to normoxia and hypoxic conditions, gCSCs were harvested, pelleted by centrifugation, and rinsed with ice-cold PBS at 1,500-rpm for 5 min. The cells were lysed for 30 min in ice-cold lysis buffer (50-mM Tris–HCl [pH 8.0], 150-mM NaCl, 1-mM EDTA) containing 1% Triton-X-100 and phosphatase and protease inhibitors (Sigma-Aldrich). The lysates were centrifuged at 14,000-rpm for 10 min at 4°C. The supernatants were collected and quantified for protein content. Equal amounts of proteins (40-µg) were electrophoretically fractionated in 8% sodium dodecyl sulfate (SDS)-polyacrylamide gels, transferred to nitrocellulose membranes, and subjected to immunoblot analysis with specific antibodies against pSTAT3 (Tyr705), total STAT3 (both are from Cell Signaling Technology, Inc., Danvers, MA), HIF-1α (Novus Biologicals, Littleton, CO) and β-actin (Sigma-Aldrich). Autoradiography of the membranes was performed using Amersham ECL Western-blotting detection reagents (Amersham Biosciences).

### Statistical analysis

All values were calculated as means and 95% confidence intervals (CIs) from at least three independent experiments. Student's *t* test was used to test for differences in the means between two groups. *P* values of less than 0.05 were considered to be statistically significant. All statistical analyses were performed using the Statistical Package for the Social Sciences v.12.0.0 (SPSS, Chicago, IL). Error bars represent the s.d.

## Results

### Hypoxia enhances the production of immunosuppressive cytokines by the gCSCs

**Figure 1:**
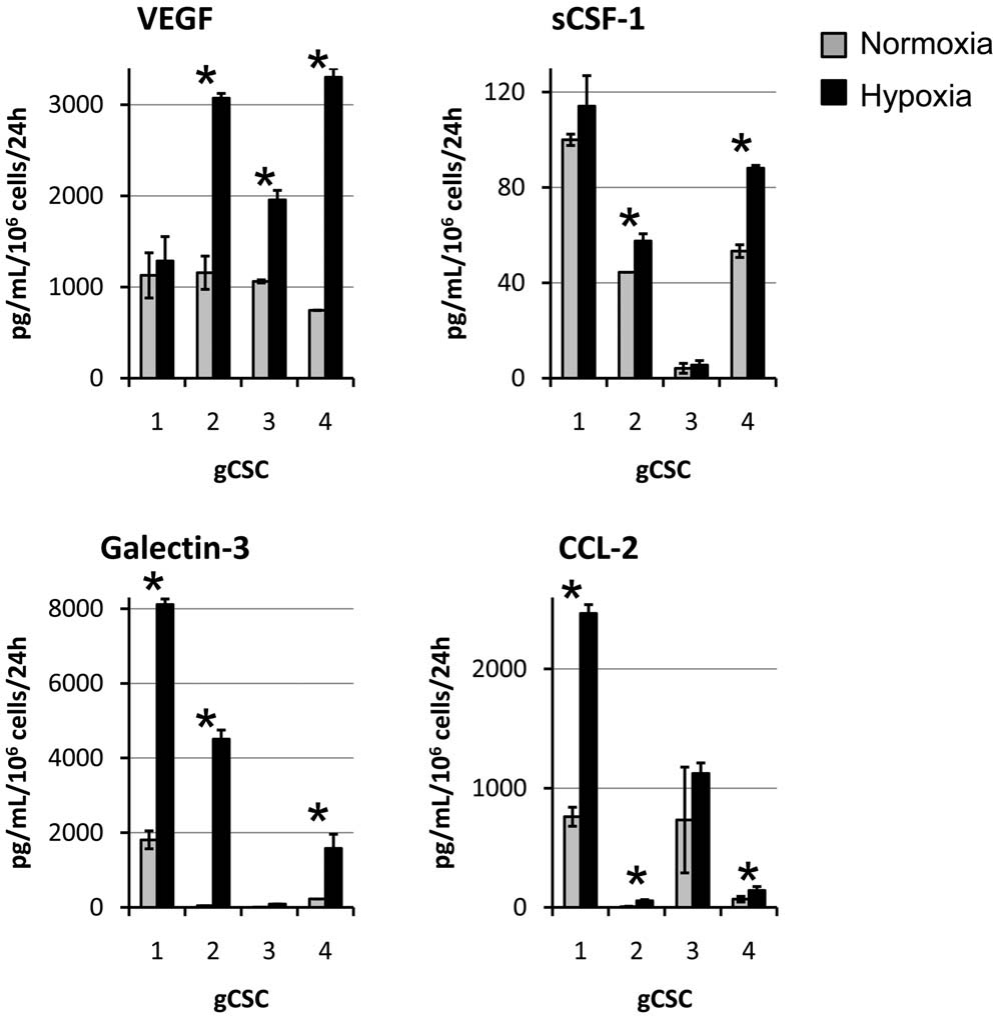
Hypoxia increased immunosuppressive cytokine production in gCSCs. Under hypoxic conditions, there was an increase in production of: VEGF in 3 of 4 gCSCs, galectin-3 in 3 out of 4 gCSCs, sCSF-1 in 3 of 4 gCSCs, and CCL-2 in 3 of 4 gCSCs, as assessed by ELISA. Error bars are derived from three separate experiments representing the deviation of the associated means between experiments throughout the manuscript. * *P*<0.05.

To determine if hypoxia further potentiated gCSC elaborated immunosuppressive cytokines, the gCSCs (n = 4) were assayed for immunosuppressive cytokines by ELISA. Hypoxia enhanced immunosuppressive VEGF secretion in 3 out of 4 gCSCs (750–1160 pg/10^6^ cells/24 hours under normoxic conditions versus 1290–3300 pg/10^6^ cells/24 hours under hypoxic conditions, an increase of 1.1–4.4X) and the monocyte chemokine attractant sCSF-1 in 2 out of 4 gCSCs (4.2–100 pg/10^6^ cells/24 hours under normoxic conditions versus 5.6–114 pg/10^6^ cells/24 hours, an increase of 1.1–1.7X). Trends were also observed showing hypoxia increasing the T cell apoptosis inducing cytokine galectin-3 secretion in 3 out of 4 gCSCs (4.2–1810 pg/10^6^ cells/24 hours under normoxic conditions versus 88.9–8110 pg/10^6^ cells/24 hours under hypoxic conditions, an increase of 4.5-101X), and the regulatory T cell chemokine attractant CCL-2 in 3 out of 4 gCSCs (5.6–761 pg/10^6^ cells/24 hours under normoxic conditions versus 56.1–2465 761 pg/10^6^ cells/24 hours under hypoxic conditions, an increase of 1.5–10.1X) (Fig. 1). Hypoxia induced variable responses in TGF-β1 production and failed to enhance production of MIC-1 (data not shown).

### Hypoxia augments the ability of gCSCs to inhibit T cell adaptive immunity

**Figure 2:**
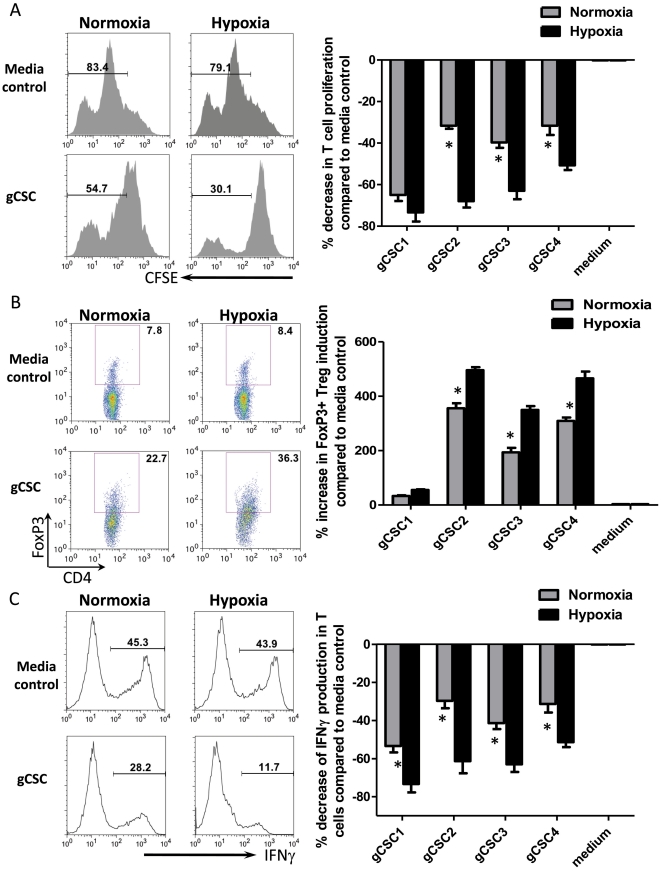
Hypoxia enhances the gCSC-mediated immunosuppression on human T cells. A. When healthy donor peripheral blood mononuclear cells (PBMCs) were cultured in the presence of supernatant medium from cultures of each of the gCSCs, T cell proliferation was inhibited, as demonstrated by fluorescence-activated cell sorting (FACS) analysis of CD3+ T cell carboxyfluorescein diacetate succinimidyl ester (CFSE) labeling that measures T cell proliferation. For each of the matched gCSCs under hypoxic conditions, T cell proliferation was further inhibited compared to normoxia. Representative FACS histograms or dot plots are shown on the left, and the summary bar graphs of percentage changes are shown on the right. T cells cultured with neurosphere cell medium served as a control. B. The supernatants from the gCSCs induced an increase in the number of FoxP3^+^ regulatory T cells in the gated CD4^+^ T cells, which is further enhanced under conditions of hypoxia. C. The inhibition of IFNγ production in CD3+ T cells was enhanced under hypoxic condition compared to normoxia. * *P*<0.05.

To determine if hypoxia potentiates the immunosuppressive effects of gCSCs on T cell activation and proliferation, the gCSCs (n = 4) were placed under both normoxic and hypoxic conditions. All supernatants from gCSCs cultured under normoxic conditions inhibited T cell proliferation by approximately 43% (range: 29–65%). Supernatants from gCSCs cultured under conditions of hypoxia further inhibited T cell proliferation represented by division of CFSE labeled T cells by approximately 64% (range: 52–78%, *P* = 0.04 comparing hypoxia to normoxia, Fig. 2A). Because hypoxia is likely to enhance the induction of regulatory T cells, we tested supernatants from gCSCs grown under normoxic conditions and found that they caused expansion of the number of CD4+FoxP3+ regulatory T cells in healthy donor PBMCs by 236% (range: 34%–400%). However, when cultured under hypoxic conditions, the gCSCs further increased the induction of regulatory T cells by 342% (range: 55%–512%, *P* = 0.05 comparing hypoxia to normoxia, Fig. 2B). To assess for functional activity of T cell suppression, IFNγ production in the CD3/CD28 stimulated T cells were measured after culture with supernatants from gCSCs exposed to normoxic and hypoxic conditions. We found that hypoxic gCSCs caused a significant decrease of the IFNγ+ T cells compared to normoxia (51–74% vs 24–53%, *P* = 0.02, Fig. 2C).

### Hypoxia enhances the ability of gCSCs to inhibit phagocytosis in monocytes

To determine whether hypoxia-stressed gCSCs would alter the expression of MHC and co-stimulatory molecules on monocytes, which give rise to macrophages, we compared monocytes that were exposed to gCSC-conditioned medium under hypoxia and normoxic conditions and then analyzed surface markers by flow cytometry. Hypoxia-stressed gCSCs did not alter the expression of CD80, CD86, MHC I, MHC II, cytotoxic T-lymphocyte antigen (CTLA)-4, or B7-H1 on monocytes in comparison with those cultured in normoxic conditions (data not shown), indicating that hypoxia did not alter the gCSC-mediated expression of inflammatory markers on the monocytes.

**Figure 3:**
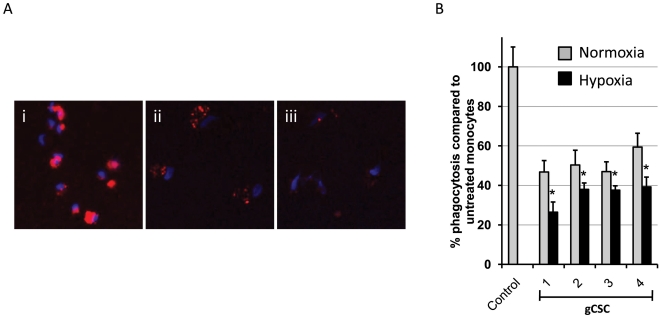
The ability of gCSCs to inhibit phagocytosis in monocytes is enhanced by hypoxic conditions. A. Supernatants from gCSCs cultured in normoxic and hypoxic conditions inhibited phagocytosis of fluorescent microbeads (red) in monocytes exposed to the supernatants for 72 h, with hypoxic supernatants inhibiting phagocytosis to a greater extent than normoxic supernatants for all 4 gCSCs tested. Representative high-power fluorescent microscope images (40X) of monocytes exposed to (i) neurosphere medium alone as a positive control, (ii) normoxic gCSC supernatant, and (iii) hypoxic gCSC supernatants. Cell nuclei were stained with DAPI (blue). B. *P<0.05 indicates a significant difference between phagocytosis inhibition by hypoxic versus normoxic gCSC supernatants.

To determine whether the ability of gCSCs to inhibit phagocytosis in monocytes was increased under hypoxic conditions, phagocytosis assays were performed on monocytes exposed to medium conditioned by gCSCs under normoxic and hypoxic conditions. Under normoxic conditions, the gCSC supernatants (n = 4) inhibited phagocytosis in monocytes (Fig. 3A) (to 47%–59% of the level seen in positive control monocytes exposed to neurosphere medium alone; *P* = 0.0005). The inhibition of phagocytosis was further enhanced to a statistically significant degree under hypoxic conditions (to 26%–39% of positive control) compared with supernatants from gCSC cultured under normoxic conditions (*P* = 0.005, paired *t*-test) in all four gCSCs tested (Fig. 3B).

### Hypoxia enhances pSTAT3 and HIF-1α activity

**Figure 4:**
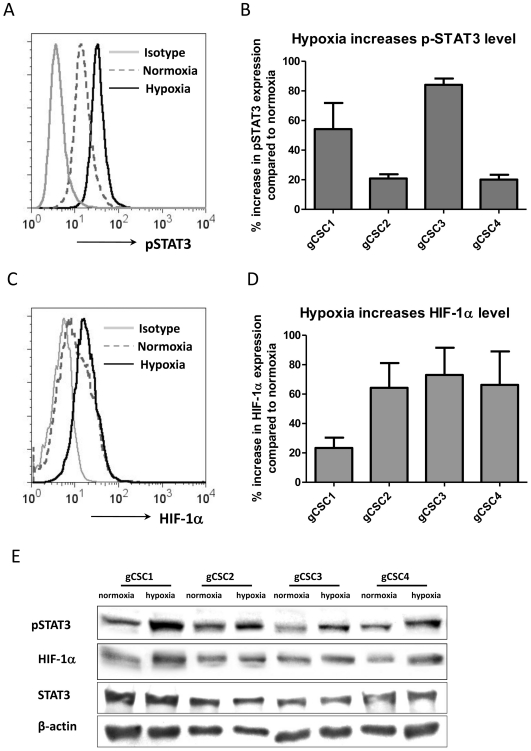
Hypoxia increased the intracellular pSTAT3 and HIF-1α expression in gCSCs. A and B. The pSTAT3 expression in the gCSCs was measured by intracellular pSTAT3 (pY705) staining via flow cytometry. The mean fluorescent intensity of positive staining was determined by flow cytometry analysis under both normoxic and hypoxic conditions for each gCSC (representative histogram was shown as A), and then the percentage change was calculated compared with the expression of pSTAT3 in each gCSC under normoxic condition (shown as B). C and D. The HIF-1α expression in the gCSCs was measured by intracellular HIF-1α staining via flow cytometry after culture in either hypoxic or normoxic culture conditions: representative histogram was shown as B, and percentage changes of all 4 gCSCs under hypoxia compared to normoxic condition was plotted as bar graphs in D. E. Confirmatory representative western blot demonstrating that pSTAT3 and HIF-1α expression is increased within gCSCs after hypoxia exposure. There was no change for total STAT3 expression.

To ascertain if hypoxia increases gCSC expression of pSTAT3 and HIF-1α, the gCSCs were exposed to hypoxia and normoxic conditions and the expression levels of pSTAT3 and HIF-1α were determined by flow cytometry analysis. Hypoxia augmented the expression of both pSTAT3 (Fig. 4A and B) and HIF-1α (Fig. 4C and 4D) by 19∼87% (average 43%) and 28∼86% (average 62%), respectively. Western blot analysis showed a similar increase of pSTAT3 and HIF-1α expression, and no change for total STAT3 (Fig. 4E), indicating the increase of pSTAT3 were not due to general STAT3 changes.

### WP744 and WP1066 affect pSTAT3 and HIF-1α activity

**Figure 5:**
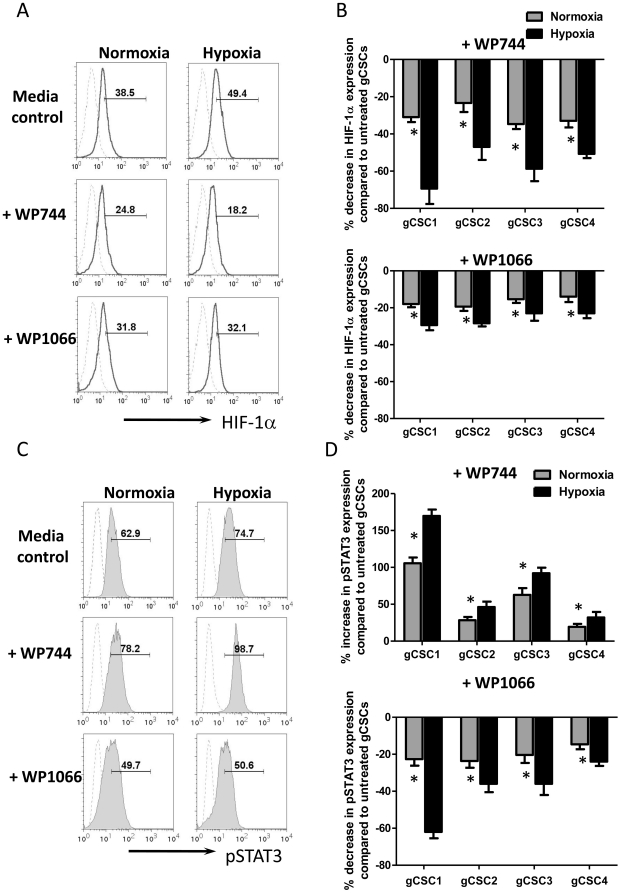
WP1066 inhibits pSTAT3 and HIF-1α expression, whereas WP744 only inhibits HIF-1α expression in gCSCs. A and B. WP744 or WP1066-treated, hypoxia and normoxia exposed gCSCs express lower levels of HIF-1α than gCSCs exposed to hypoxia and normoxia alone. The HIF-1α expression in the gCSCs cultured under normoxia and hypoxic conditions was measured by intracellular staining via flow cytometry in the presence or absence of WP744 or WP1066. Representative histograms were shown as A. The percentage changes of all 4 gCSCs under hypoxia and normoxia were compared to untreated gCSCs and then the corresponding percentage change of expression was calculated and plotted as bar graphs in B. C and D. WP1066 inhibited pSTAT3 expression in gCSCs under both normoxia and hypoxia, whereas WP744 enhances pSTAT3 expression. The pSTAT3 expression in the gCSCs under normoxia and hypoxic conditions was measured by intracellular pSTAT3 staining via flow cytometry in the presence or absence of WP744 or WP1066. Representative histograms were shown as C, and percentage changes of all 4 gCSCs under hypoxia and normoxia were plotted as bar graphs in D. In histograms, dashed line represented isotype staining and the black line staining for either HIF-1α or pSTAT3. The number in histograms represented positive percentage for pSTAT3 or HIF-1α relative to the isotype. *P<0.05.

To determine if we could reverse the hypoxia-induced immunosuppression, we first assessed the ability of WP744 and WP1066 to reverse hypoxia-induced expression of HIF-1α. WP744 was a more potent inhibitor of HIF-1α and reduced expression by 53% under conditions of hypoxia, compared to 25% HIF-1α reduction by WP1066 (Figure 5A and 5B), thereby essentially abolishing the hypoxia-induced amounts of HIF-1α A similar but less degree inhibition of HIF-1α was observed under conditions of normoxia, confirming that these agents possessed HIF-1α inhibitory properties. However, we found WP744 enhanced pSTAT3 expression, but WP1066 inhibited its expression under both conditions of normoxia and hypoxia (Figure 5C and 5D). There were up-regulation of pSTAT3 expression by 25%–184% (*P* = 0.02, paired *t*-test) in all 4 gCSCs under hypoxia (Fig. 5D), in contrast to the inhibitory effect of WP1066 (Fig. 5D) (decrease 24%–68% under hypoxia alone, *P* = 0.0005, paired *t*-test), indicating that WP1066 possess inhibitory activity of STAT3 and HIF-1α, whereas WP744 only inhibits HIF-1α.

### Inhibition of pSTAT3 and HIF-1α reverses hypoxia induced immunosuppression

To assess the impact of WP1066 and WP744 on cytokine secretion in hypoxia-exposed gCSC, the gCSCs were treated with each inhibitor under conditions of hypoxia. WP1066 reduced galectin-3 (43%–79%, *P* = 0.02) and sCSF-1 (0%–24%, *P* = 0.05) production in all four hypoxia-exposed gCSCs, VEGF production in 3/4 gCSCs (23%–67%, *P* = 0.08), and CCL-2 production in 2/4 gCSCs (16%–46%, *P* = 0.04). The gCSCs in which WP1066 did not affect CCL-2 production (gCSC2 and gCSC4) produced only very low levels of CCL-2 under either normoxic or hypoxic conditions. WP744 reduced galectin-3 (79%–98%, *P* = 0.09) in all four gCSCs, and VEGF in 3/4 gCSCs (23%–41%, *P* = 0.04), but increased sCSF-1 production (105%–159%, *P* = 0.05), and had no significant impact on CCL-2 production (data not shown). In gCSC1, WP1066 and WP744 did not affect VEGF production; but hypoxia had the least substantial effect on VEGF production relative to normoxia in gCSCs (Fig. 1).

**Figure 6:**
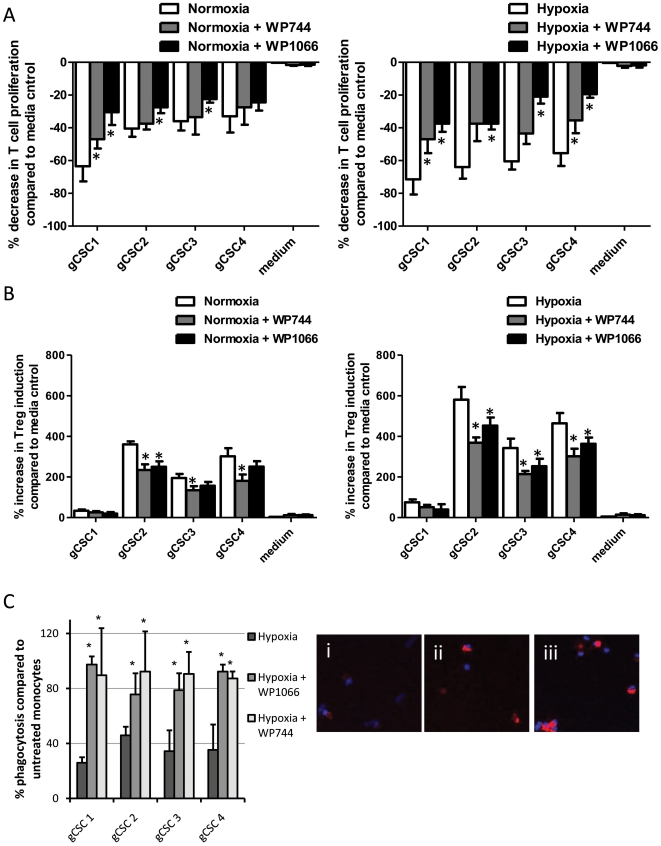
Inhibition of pSTAT3 and HIF-1α reverses hypoxia-gCSCs induced immunosuppression A. WP1066 reverses gCSC-mediated immunosuppression in human T cells to a greater degree than WP744. When healthy donor PBMCs were cultured in the presence of each of the supernatants conditioned by the gCSCs, T cell proliferation was inhibited, as demonstrated by FACS analysis on CFSE labeled T cell division. This inhibitions was further potentiated by hypoxia as previous shown but could be reversed with WP1066 to the greater degree than WP744. B. The supernatant culture mediums from the gCSCs induces an increase in the number of FoxP3^+^ regulatory T cells among the gated CD4^+^ T cells that is further enhanced under conditions of hypoxia as previously shown. This was reversed with WP744 to a greater degree than with WP1066. The controls were T cells cultured in respective neurosphere medium of normoxia and hypoxic conditions. * *P*<0.05 versus untreated gCSCs exposed to normoxia or hypoxia C. Both WP1066 and WP744 reversed the inhibition of phagocytosis in monocytes by hypoxia exposed gCSCs in all 4 gCSCs. Shown are representative high-power fluorescent microscope images (40X) of monocytes cultured with (i) hypoxia-exposed gCSC supernatant, (ii) WP1066-treated, hypoxia-exposed gCSC supernatant, and (iii) WP744-treated, hypoxia-exposed gCSC supernatant. * *P*<0.05 versus untreated hypoxia-exposed gCSCs.

We next addressed the potency of these agents in reversing hypoxia-exposed gCSC-mediated immunosuppression. At physiological levels that can be obtained *in vivo*, WP1066 was more potent than WP744 at restoring T cell proliferative responses (Fig. 6A). In contrast, WP744 was able to inhibit the hypoxia-induced CD4+FoxP3+ T cell to a greater degree than WP1066 (Fig. 6B). WP1066 and WP744 were equally effective in restoring monocyte phagocytosis inhibition (Fig. 6C). WP1066 increased phagocytosis to 86.0% in comparison to neurosphere medium positive control (range 75.6% to 97.3%, *P* = 0.01) and WP744 increasing phagocytosis to 90.0% (range 87.3% to 92.3%, *P* = 0.0006).

## Discussion

In this investigation we demonstrated that tumor hypoxia potentiates glioma-mediated immunosuppression of both innate and adaptive immunity. We had previously shown that the gCSCs induced regulatory T cells [3]; however, during blocking experiments of TGF-β, we could not completely ablate the induction of regulatory T cells (unpublished data) indicating that other cytokines had a role in triggering the formation of regulatory T cells. Under conditions of hypoxia, there was a marked increase of HIF-1α by the gCSCs. A previous report has demonstrated that hypoxia can increase HIF-1α levels and subsequent FoxP3 expression in T cells along with their corresponding potency in suppressing effector T cells [10]. It has been proposed by Sitkovsky et al., [44] that maximal immunosuppression is only possible if the T cell receptor (TCR)-triggered activities of immunosuppressive regulatory T cells are combined with and/or enhanced by tissue hypoxia via the adenosine receptor [45]. Our studies support this contention because T cell proliferation and the induction of regulatory T cells were potentiated in hypoxic conditions. Macrophages have previously been demonstrated in hypoxic areas of tumors [46], [47], [48] likely recruited by tumor-elaborated factors [49], [50]. However, in contrast to other investigators who found that hypoxia directly enhanced monocyte/macrophage phagocytosis [51], [52], [53], we found that hypoxic exposed gCSCs inhibited monocyte/macrophage phagocytosis emphasizing the importance of the tumor microenvironment and cell-to-cell interactions in determining immunological responses.

The role of hypoxia on subverting macrophages to TAM/M2 phenotype and function likely involves both HIF-1α-dependent and independent processes. Hypoxic conditions increased the ability of gCSCs to induce pSTAT3 expression in monocytes – a defining characteristic of the M2 macrophage [54]. However, this did not result in an increase of monocyte/macrophage-elaborated immunosuppressive cytokines or further reduction in the expression of co-stimulatory and MHC molecules needed for antigen presentation on the monocytes/macrophages, indicating that hypoxia is not solely responsible for polarizing the monocytes toward a functionally immunosuppressive phenotype. It's also possible that after monocytes are freshly recruited into hypoxic areas of tumor, their further polarization towards M2 and otherwise tumor-supportive macrophage phenotypes (including the induction of immune suppressive and/or pro-angiogenic cytokines) may be directly mediated by the hypoxic environment itself [55], in addition to the direct activities of gCSCs. For example, although we did not observe induction of VEGF production in monocytes exposed to the supernatants from either normoxic or hypoxia-exposed gCSCs, VEGF production in macrophages has been shown to be induced directly by hypoxia [56].

In practice, we are exploiting the gCSCs as an immunosuppressive cell population to evaluate the role of hypoxia given the gCSCs propensity to recapitulate many of the hallmark features of malignant gliomas, including immunosuppression; however it is unlikely that hypoxia exerts effects exclusively to this population. Hypoxia induced an increase in the production of the immunosuppressive cytokines by the gCSCs such as galectin-3 and VEGF, regulatory T cell and macrophage chemoattractants sCSF-1 [19] and CCL-2 [21], [57], and HIF-1α [10] but the gCSC is unlikely to be the sole source of these cytokines within the tumor microenvironment. It has also been suspected that hypoxia may influence the levels or activity of CTLA-4 and/or B7-H1 [58]. Although we did not find differences in the expression of CTLA-4 or B7-H1 under conditions of hypoxia, we can't discount the possibility of alterations in the transcriptional activities of these receptors.

To determine whether STAT3 and HIF-1α inhibitors can reverse the hypoxia-exposed gCSC-mediated immunosuppressive effects, we treated the gCSCs with WP744 and WP1066 under hypoxic conditions. WP1066 in comparison to WP744 had a more potent effect on reduction of immunosuppressive cytokines such as galectin-3, CCL-2 and sCSF-1 elaborated by the gCSCs under hypoxia likely secondary to WP1066 inhibiting the immune suppressive pathway pSTAT3. Both of these agents were capable of inhibiting HIF-1α, with WP744 being more potent. The immunological effects of inhibiting HIF-1α were most evident in the inhibition of induced FoxP3+ T cells in which WP744 exerted the greatest effect. However, WP1066, which also exert potent STAT3 inhibitor activity, exerted a greater and more global reversal of gCSC-mediated immunosuppression on effector responses, especially adaptive T cell proliferation and effector function, although this was not absolute. This indicates that hypoxia is a significant inducer of gCSC-mediated immunosuppression but that this can't be overcome by HIF-1α inhibitors alone or that the current generation of STAT3 inhibitors can completely restore immunological responsiveness to the levels seen in normal control. Further investigations will be necessary to ascertain how to optimize the STAT3 inhibitors in combination with other immune therapeutics in the setting of hypoxic tumor microenvironments.

### Conclusion

This study demonstrates that hypoxia increases the ability of gliomas to induce immunosuppressive cells such as Tregs and M2 macrophages and thus plays a key role in both tumor-mediated innate and adaptive immune suppression. These findings also help to reconcile the disparate findings that immune therapeutic approaches can be used successfully in preclinical model systems but have yet to achieve successful responses in the vast majority of patients by demonstrating the importance of the tumor hypoxic environment in markedly inducing the immune suppressive properties of the GBM. The tumor hypoxia induced immune suppression is modulated by the signal transducer and activator transcription 3 (STAT3) pathway and hypoxia inducible factor (HIF)-1α, which can be reversed with STAT-3 and HIF-1α inhibitors and indicates hypoxia-mediated immune suppression can be overcome with inhibitors. These agents should be included in the armamentarium of agents to reverse tumor-mediated immune suppression. These findings contribute to understanding the mechanisms that impact tumor-mediated immunosuppression and provides an approach for optimizing immune therapeutic strategies.
